# Brain metabolism and neurological symptoms in combined malonic and methylmalonic aciduria

**DOI:** 10.1186/s13023-020-1299-7

**Published:** 2020-01-22

**Authors:** Sara Tucci

**Affiliations:** Department of General Pediatrics and Adolescent Medicine, Laboratory of Clinical Biochemistry and Metabolism, Faculty of Medicine, Medical Center – University of Freiburg, University of Freiburg, Mathildenstrasse 1, 79106 Freiburg, Germany

**Keywords:** Combined malonic and methylmalonic aciduria, ACSF3, mtFASII, Metabolic flexibility, Brain energy metabolism

## Abstract

Combined malonic and methylmalonic aciduria (CMAMMA) is an inborn error of metabolism which has been proposed being a benign condition. However, older patients may present with neurological manifestations such as seizures, memory problems, psychiatric problems and/ or cognitive decline. In fibroblasts from CMAMMA patients we have recently demonstrated a dysregulation of energy metabolism with increased dependency on β-oxidation for energy production. Because of the inability of the brain to rely efficiently on this pathway to retrieve the required energy to a great extent, we hypothesize an alternative disease-causing mechanism that does not only include the accumulation of the metabolites malonic and methylmalonic acids. Here, we suggest a novel hypothesis on the possible pathophysiological mechanism responsible for the development of neurological symptoms in the long-run.

Combined malonic and methylmalonic aciduria (CMAMMA) is an inborn error of metabolism caused by deficiency of the mitochondrial enzyme malonyl-CoA synthetase encoded by *ACSF3* [1]. This enzyme catalyzes the enzymatic activation of malonic acid to malonyl-CoA which is the first step of the mitochondrial fatty acid biosynthesis (mtFASII) pathway. A cross-sectional multicenter retrospective study identified 25 patients with favorable clinical course strongly suggestive of the benign condition of CMAMMA [2]. On the other hand, this disease was associated in the past with a wide range of neurological symptoms including seizures, memory problems, psychiatric problems and/ or cognitive decline especially in older patients [3–6]. Despite few reports describing symptomatic CMAMMA patients [6, 7], the clinical significance of this disease remains controversial [2]. So far, the mechanism of symptoms development has not yet been elucidated, although the involvement of the accumulating metabolites malonic acid (MA) and methylmalonic acid (MMA) has been proposed. Very recently our work on the comprehensive metabolic phenotyping of fibroblasts from CMAMMA patients demonstrated a deeply altered metabolic flexibility. This was characterized by a reduced mitochondrial respiration and glycolytic flux due to a lower lipoylation degree as well as by the reduction of anaplerotic amino acids to address very likely the required energy need [8]. Of particular importance was the finding that the dysregulation of mitochondrial energy metabolism was accompanied by the compensatory increased dependency on β-oxidation for energy production [8]. Focusing on this special point, we here propose a new hypothesis on the possible long-term mechanism of neurological symptoms associated with this disorder.

A similar phenotype to CMAMMA does appear in a very recently described new disorder of mtFASII pathway, namely MEPAN (mitochondrial enoyl CoA reductase protein-associated neurodegeneration) [9]. This neurodegenerative disease may present with childhood-onset dystonia, optic atrophy, and basal ganglia signal abnormalities, whereas intellectual abilities may remain unaffected [9]. The symptoms mimic mitochondrial diseases by the involvement of organs with high energy demand and an overall high susceptibility to oxidative stress [9]. In contrast to MEPAN, lipoylation degree is not uniformly reduced in all analyzed ACSF3 fibroblasts [8, 9]. The mitochondrial ACC1 isoform 1 in mammalian cell is able to cover in part the activation to malonyl-CoA in case of deficient ACSF3 [10] a process that may also explain the wide heterogeneous clinical phenotype described for CMAMMA. Our data in fibroblasts suggest a role of mtFASII in the regulation of energy homeostasis [8], although this can be extremely variable as it may depend on the energy need and the ability to adapt which is organ and tissue specific.

With special regard to neural cells, despite the high energy demand [11], they are not able to rely efficiently on the degradation of fatty acids for energy production to a great extent [12]. With the exception for specialized hypothalamic neurons [13], the oxidation of fatty acid with the specific purpose of energy production occurs exclusively in glial cells [14] although a tight metabolic cooperation between neurons and astrocyte is required to maintain cellular functionality [15, 16]. From an evolutionary point of view, it has been suggested that the disadvantage of the biochemical process of degradation of fatty acids has driven the pressure to promote glucose oxidation in the brain [17]. Indeed, the degradation of a molecule of palmitate requires higher oxygen consumption than oxidizing a molecule of glucose, thus avoiding the risk of hypoxia that limits the regeneration of ATP by mitochondria [17–19]. Moreover, enhanced β-oxidation is also linked to the generation of superoxides and oxidative stress [20, 21]. Our data on CMAMMA fibroblasts clearly demonstrated a shift towards β-oxidation for energy production, a biochemical finding associated to a reduction of respiratory complexes I to III and an increase of cardiolipin species [8]. Although our results cannot be automatically translated to neural cells under physiological conditions, we may speculate that a possible compensative/ adaptive upregulation of fatty acid degradation may occur in brain cells. Our hypothesis is that the chronic and latent upregulation of mitochondrial β-oxidation with the subconsequent increment of risk for hypoxia and oxidative stress in CMAMMA patients may be crucial for the onset of neurological symptoms in the long-run.

Due to the key role of mtFASII pathway on metabolic flexibility and cellular energy maintenance in fibroblasts and neural cells [8, 9], it is conceivable to assume an upregulation of β-oxidation in case of hypofunctional mtFASII also in brain cells. We may speculate that the long-term stimulation of fatty acid oxidation may be counterproductive and increase the risk for hypoxia and oxidative stress in a chronic and latent manner Fig. 1. This effect together with additional variables such as increased levels of MA and MMA and environmental factors may lead in some patients to the onset of neurological symptoms in the long run. Long-term studies in mouse model of ACSF3 deficiency and human iPS-derived cell lines will be critical to support the role of mtFASII in mammalian systems.

**Fig. 1:**
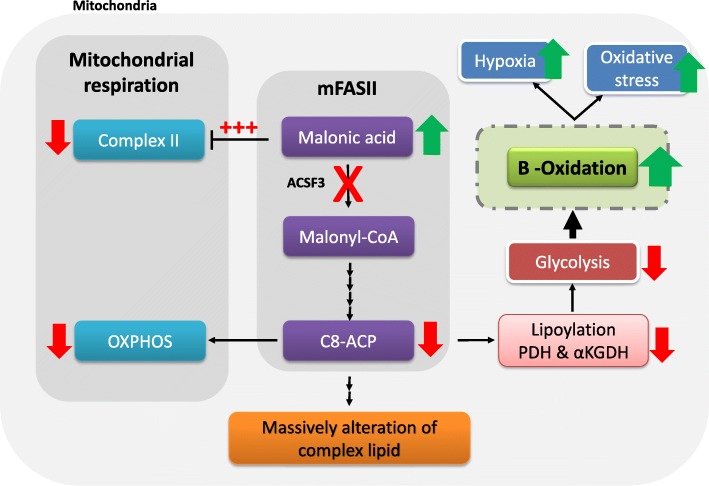
Schematic representation of the compensatory/ adaptive mechanisms of upregulation of mitochondrial β-oxidation in response to hypofunctional mtFASII pathway due to mutations in ACSF3 gene. Figure modified and adapted from [8]

## Data Availability

The datasets supporting the conclusions of this article are included in the cited article “The emerging role of the mitochondrial fatty-acid synthase (mtFASII) in the regulation of energy metabolism “published by the same author (Wehbe et al., Biochim Biophys Acta Mol Cell Biol Lipids. 2019 Jul 31;1864(11):1629–1643, PMID: 31376476).

## References

[1] Witkowski A, Thweatt J, Smith S (2011). Mammalian ACSF3 protein is a malonyl-CoA synthetase that supplies the chain extender units for mitochondrial fatty acid synthesis. J Biol Chem.

[2] Levtova A, Waters PJ, Buhas D, Levesque S, Auray-Blais C, Clarke JTR, Laframboise R, Maranda B, Mitchell GA, Brunel-Guitton C (2019). Combined malonic and methylmalonic aciduria due to ACSF3 mutations: benign clinical course in an unselected cohort. J Inherit Metab Dis.

[3] de Sain-van der Velden MG, van der Ham M, Jans JJ, Visser G, Prinsen HC, Verhoeven-Duif NM, van Gassen KL, van Hasselt PM (2016). A new approach for fast metabolic diagnostics in CMAMMA. JIMD Rep.

[4] Pupavac M, Tian X, Chu J, Wang G, Feng Y, Chen S, Fenter R, Zhang VW, Wang J, Watkins D (2016). Added value of next generation gene panel analysis for patients with elevated methylmalonic acid and no clinical diagnosis following functional studies of vitamin B12 metabolism. Mol Genet Metab.

[5] Reid ES, Papandreou A, Drury S, Boustred C, Yue WW, Wedatilake Y, Beesley C, Jacques TS, Anderson G, Abulhoul L (2016). Advantages and pitfalls of an extended gene panel for investigating complex neurometabolic phenotypes. Brain.

[6] Sloan JL, Johnston JJ, Manoli I, Chandler RJ, Krause C, Carrillo-Carrasco N, Chandrasekaran SD, Sysol JR, O'Brien K, Hauser NS (2011). Exome sequencing identifies ACSF3 as a cause of combined malonic and methylmalonic aciduria. Nat Genet.

[7] Alfares A, Nunez LD, Al-Thihli K, Mitchell J, Melancon S, Anastasio N, Ha KC, Majewski J, Rosenblatt DS, Braverman N (2011). Combined malonic and methylmalonic aciduria: exome sequencing reveals mutations in the ACSF3 gene in patients with a non-classic phenotype. J Med Genet.

[8] Wehbe Z, Behringer S, Alatibi K, Watkins D, Rosenblatt D, Spiekerkoetter U, Tucci S (2019). The emerging role of the mitochondrial fatty-acid synthase (mtFASII) in the regulation of energy metabolism. Biochim Biophys Acta Mol Cell Biol Lipids.

[9] Heimer G, Keratar JM, Riley LG, Balasubramaniam S, Eyal E, Pietikainen LP, Hiltunen JK, Marek-Yagel D, Hamada J, Gregory A (2016). MECR mutations cause childhood-onset dystonia and optic atrophy, a mitochondrial fatty acid synthesis disorder. Am J Hum Genet.

[10] Monteuuis G, Suomi F, Keratar JM, Masud AJ, Kastaniotis AJ (2017). A conserved mammalian mitochondrial isoform of acetyl-CoA carboxylase ACC1 provides the malonyl-CoA essential for mitochondrial biogenesis in tandem with ACSF3. Biochem J.

[11] Watts ME, Pocock R, Claudianos C (2018). Brain energy and oxygen metabolism: emerging role in Normal function and disease. Front Mol Neurosci.

[12] Romano A, Koczwara JB, Gallelli CA, Vergara D, Micioni Di Bonaventura MV, Gaetani S, Giudetti AM (2017). Fats for thoughts: an update on brain fatty acid metabolism. Int J Biochem Cell Biol.

[13] Wolfgang MJ, Cha SH, Millington DS, Cline G, Shulman GI, Suwa A, Asaumi M, Kurama T, Shimokawa T, Lane MD (2008). Brain-specific carnitine palmitoyl-transferase-1c: role in CNS fatty acid metabolism, food intake, and body weight. J Neurochem.

[14] Panov A, Orynbayeva Z, Vavilin V, Lyakhovich V (2014). Fatty acids in energy metabolism of the central nervous system. Biomed Res Int.

[15] Belanger M, Allaman I, Magistretti PJ (2011). Brain energy metabolism: focus on astrocyte-neuron metabolic cooperation. Cell Metab.

[16] Pellerin L, Magistretti PJ (2012). Sweet sixteen for ANLS. J Cereb Blood Flow Metab.

[17] Schonfeld P, Reiser G (2013). Why does brain metabolism not favor burning of fatty acids to provide energy? Reflections on disadvantages of the use of free fatty acids as fuel for brain. J Cereb Blood Flow Metab.

[18] Erecinska M, Silver IA (1989). ATP and brain function. J Cereb Blood Flow Metab.

[19] Siesjo BK (1978). Brain energy metabolism and catecholaminergic activity in hypoxia, hypercapnia and ischemia. J Neural Transm Suppl.

[20] Schonfeld P, Wojtczak L (2008). Fatty acids as modulators of the cellular production of reactive oxygen species. Free Radic Biol Med.

[21] Waldbaum S, Patel M (2010). Mitochondria, oxidative stress, and temporal lobe epilepsy. Epilepsy Res.

